# The Role of Hydrogeography and Climate in the Landscape Epidemiology of West Nile Virus in New York State from 2000 to 2010

**DOI:** 10.1371/journal.pone.0030620

**Published:** 2012-02-06

**Authors:** Michael G. Walsh

**Affiliations:** Department of Epidemiology and Biostatistics, School of Public Health, State University of New York, Downstate, New York, New York, United States of America; Kenya Medical Research Institute - Wellcome Trust Research Programme, Kenya

## Abstract

The epidemiology and ecology of West Nile virus (WNV) have not yet been completely described. In particular, the specific roles of climate and water in the landscape in the occurrence of human WNV cases remain unknown. This study used Poisson regression to describe the relationships between WNV cases and temperature, precipitation, and the hydrogeography of the landscape in New York State from 2000 to 2010. Fully adjusted models showed that hydrogeographic area was significantly inversely associated with WNV cases (incidence rate ratio (IRR) = 0.99; 95% C.I. = 0.98–0.997, p = 0.04), such that each one square kilometer increase in hydrogeographic area was associated with a 1% decrease in WNV incidence. This association was independent of both temperature, which was also associated with WNV incidence (IRR = 2.06; 95% C.I. = 1.84–2.31, p<0.001), and precipitation, which was not (IRR = 1.0; 95% C.I. = 0.99–1.01, p = 0.16). While the results are only suggestive due to the county-level aggregated data, these findings do identify a potentially important surveillance signal in the landscape epidemiology of WNV infection.

## Introduction

West Nile virus (WNV) was introduced into the United States during the summer season of 1999 in New York City. The virus was able to establish itself in each of the 48 contiguous states by 2005. Indeed, all the contiguous states other than Oregon and Washington were reporting human cases by the 2003 season [1]. This virus has disseminated throughout North America with extraordinary swiftness [2], and has contributed to multiple and ongoing local and regional epidemics, which is in contrast to the stable endemicity in Africa and southern Asia, or sporadic occurrence in Europe [2]. While *Culex* mosquitoes and passerine birds have been fairly well established as the primary vectors and reservoir hosts, respectively [3], important aspects of the landscape epidemiology of WNV remain unknown. Moreover, in New York State WNV epidemics had given way to WNV endemicity within only a few years after its introduction. While some evidence is building regarding the key mechanisms of such rapid distribution, open questions remain, particularly with respect to virus transmission in the context of certain geographic phenomena. For example, there is now some evidence that the primary *Culex* vectors are different east and west of the Mississippi River, with *Culex pipiens* being the primary vector in the east, and *C. tarsalis* being the primary vector in the west [4]–[7]. In addition, both precipitation and temperature seem to play pivotal roles in WNV ecology, which is to be expected given that 1) mosquitoes require water for ovipositioning and 2) higher temperatures shorten the time required to complete their life cycle. Higher precipitation and temperature have both been associated with increased WNV transmission in the United States [8]. Interestingly, however, low precipitation in concert with high temperature has also been associated with greater incidence of WNV infection in humans [9], [10]. Reasons for the latter inverse relationship between low precipitation and increased human WNV cases are not clear, but may be due to more widespread dispersal of the mosquitoes' preferred hosts, passerine birds, during periods of local drought [11]. Land cover has also been used to delineate geographic differences in WNV transmission. It has been suggested that land use differences between the eastern and western United States correspond to differences in WNV ecology. These differences are purported to reflect differences in habitat preferences between the two primary WNV vectors, respectively, *C. pipiens* in the east and *C. tarsalis* in the west [12]. Nevertheless, we still understand little about the extraordinarily complex landscape epidemiology of WNV. In particular, the distribution of water across the landscape, i.e. the hydrogeography, may be of particular importance in the distribution of WNV [13]–[15]. Moreover, the role of hydrogeography in concert with other climate factors, such as precipitation and temperature, have not been adequately described as a component of WNV epidemiology and disease transmission. Nor have these aspects of the physical landscape been considered in combination with WNV surveillance in birds, which could be quite useful given that passerine birds are both the reservoir for WNV and the preferred hosts of *C. pipiens*. As such, these birds are fundamental to the disease ecology of WNV in the northeastern part of North America [3], [16], and may be important in other areas of North America as well [17]. This paper seeks to take a hydrogeographic approach to human WNV occurrence by combing data on surface water with climate data during each of the years of WNV transmission, from 2000 to 2010, in the 62 counties of New York State. This investigation hypothesized that the hydrogeography, specifically the total area represented by large bodies water, would exhibit a negative association with WNV infection in humans independent of the weather. Secondarily, it was hypothesized that WNV infections in birds in these same counties may have the potential to mediate the association between water in the landscape and human infection with WNV.

## Methods

All incident cases of WNV in humans were obtained from the New York State Department of Health by county for each of the years from 2000 through 2010 [18]. In addition, surveillance of WNV in dead birds was also recorded in each county from 2000 through 2008. NYS DOH identifies human WNV cases by an ongoing surveillance system, which was initially adopted during and after the 1999 epidemic in New York City [19]. Briefly, New York State's Department of Health relied on the Health Information Network during the initial outbreak to identify cases of WNV disease. Subsequently, in preparation for sustained transmission in the following season, the Department developed a surveillance program that combined the monitoring of mosquitoes, bird populations, and human cases. The coordinated actions of the Health Information Network and the newly developed species monitoring served as the foundation for the subsequent decade of WNV surveillance in New York State [19].

Climate data were obtained from a monthly surface data product from the National Climate Data Center, which manages meteorologic data products for the National Oceanic and Atmospheric Administration [20]. Daily recordings of precipitation and temperature were not available from this agency, thus monthly recordings provide the finest resolution available. Total monthly precipitation for each county for each year in New York State was obtained and summed over the six month period from May through October representing the mosquito and WNV transmission seasons. Seasonal precipitation appeared to demonstrate greater relevance for WNV occurrence than did annual precipitation. Using the total accumulated precipitation over 12 months, there was no crude association between annual precipitation and human infection (IRR = 1.0, 95% C.I. 0.99–1.0, p = 0.46) and no crude association between annual precipitation and bird infection (IRR = 1.0, 95% C.I. 0.99–1.0, p = 0.39). Moreover, replacing seasonal precipitation with annual precipitation in the multiple logistic regression model did not quantitatively or qualitatively change the association between hydrogeographic area and human infection, or between temperature and human infection. Moreover, this was similar to another report that failed to identify any association between annual precipitation and human WNV infection [17]. Based on the data under current investigation, as well as their agreement with previously published work, it was decided to use seasonal precipitation rather than annual precipitation.

Mean monthly temperatures were also recorded for each of the 11 years from 2000 through 2010. Given the obvious problem of temperature variation by month, but the potential residual impact of higher temperatures overall throughout the WNV season, this study took a unique approach to quantifying temperature. Rather than creating discrete panel temperature data for each month, a monthly-weighted cumulative measure of temperature was used to represent the mosquito and WNV seasons as a whole from May through October. The mean temperature for each month from May through October was multiplied by its weighting factor, which consisted of the proportion (ranging from 0 to 1) of that month's temperature's estimated contribution to the overall occurrence of WNV in humans throughout the season. The relative importance of each month's temperature to the collective season's WNV occurrence was determined by the average distribution of heat across each month from May through October. Hotter months were weighted higher because of their relative importance for mosquito development and for WNV transmission. The specific weighting is as follows: the mean temperature for May was multiplied by 0.05, the mean temperature for June was multiplied by 0.15, and July, August, September, and October mean temperatures were multiplied by 0.3, 0.3, 0.15, and 0.05, respectively, and are depicted in the equation below:
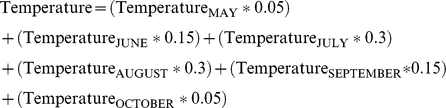
These weights reflect the relative importance considered for each month's mean temperature to the overall occurrence of WNV for each year. The weighted mean temperatures for each month were then summed over all six months to create a single seasonal temperature for each county for each year under study, which then has the same unit interpretation in degrees Celsius. To test the validity of this seasonal temperature measure, sensitivity analyses were conducted wherein the analyses were repeated using the mean temperature for each month alone, again from May through October. The associations between temperature and WNV, and the temperature-adjusted associations between precipitation and hydrogeographic area and WNV, were very similar between the seasonal temperature measure and the individual monthly mean temperatures. Therefore this investigation proceeded with the use of the monthly-weighted seasonal temperature, which is a more comprehensive measure of temperature, as well as being more easily interpretable on a year to year basis.

Hydrogeography data were obtained from the United States Geological Survey National Hydrology Dataset [21]. The area, in square kilometers, of all lakes, ponds, swamps, and marshes was used to designate the total surface water per county. The area per county was obtained by calculating the geometry in a geographic information system (GIS). There were no differences observed across types of hydrogeographic features, i.e. lakes and ponds versus swamps and marshes, so all freshwater bodies were combined for easier presentation and interpretation of results. Hereafter, these combined water bodies will be referred to as hydrogeographic features, and the area they represent will be referred to as the hydrogeographic area. While both precipitation and these hydrogeographic features contribute to the overall surface water present in the landscape, the distinction between them follows the assumption that the former, i.e. precipitation, defines the more transient water features in the landscape, while the latter, i.e. hydrogeography, defines the more fixed water features in the landscape.

Random effects models were fit using Poisson regression for panel data and the results used to identify the associations between the hydrogeography and climate factors and human WNV infection per county across the 11 years of WNV surveillance. These panel data are longitudinal data, wherein instances of observations can be conceptualized as discrete time points at each time, *i*. Moreover, panel data allow for both a temporal and spatial designation of the observations, thus they are fundamentally multidimensional data. With the application of random effects models, each case at time *i* and space *j* can be considered as the outcome of a random process. Poisson regression models count data, which are assumed to follow a Poisson distribution. This is a probability distribution of events observable in discrete intervals of time and space. In the current study, the Poisson regression is modeling the annual counts of human infections per the total county population, and therefore estimates the incidence rate. As such, incident rate ratios are reported in the results. Total seasonal precipitation, seasonal temperature, and hydrogeographic area all demonstrated bivariate associations with WNV cases and were subsequently included in an adjusted Poisson model as independent variables. The number of WNV-infected dead birds each year was also associated with the number of human infections per county. An additional aim of this study was to explore whether the associations between climate and hydrogeography factors and human WNV cases may be mediated by the extent of bird infections per year. Mediation cannot be adequately assessed simply by adding the purported mediator to a regression model and examining the change in regression coefficients of the model predictors whose effects are hypothesized to be mediated. To more appropriately assess the potential mediation of climate and hydrogeography by bird infection, instrumental variable regression was applied with hydrogeographic area and precipitation as the specified instruments, once again in regression analysis that accounted for the county and time panels inherent in the data.

Finally a map was constructed to show the spatial fit of hydrogeography and climate factors as predictors of WNV cases. A simple approach was adopted by adding just two layers to the map. The first layer is a monochrome choropleth of the predicted cumulative incidence of WNV from 2000 to 2010. The second layer overlays the choropleth with graduated symbols representing the actual number of observed cases per county over the same 11 year period.

ArcGIS was used for all mapping procedures and for calculating the Moran's Index to identify whether spatial clustering of the residuals was present (ESRI), and STATA v.11 was used for the Poisson and instrumental variable regression modeling, both specific to panel data, with the *xtpoisson* and *xtivreg* command procedures, respectively (STATA Corp., College Stattion, TX).

## Results

During the 11 year period from 2000 to 2010, 457 human cases of WNV occurred across the state of New York. These cases were distributed across just 29 of 62 counties (47%), ranging from 1 to 128 cumulative incident cases per county. New York State has a cumulative surface water area of 2619 square kilometers. The median hydrogeographic area across all counties is 40 square kilometers, but these counties also demonstrated considerable hydrogeographic variability, ranging from less than two square kilometers to 421 square kilometers.

Table 1 presents crude bivariate associations between WNV cases and hydrogeographic area, precipitation, and temperature each separately. The first model in Table 1 shows the crude association between total precipitation and WNV cases. The incidence rate ratio (IRR) is 0.98 (95% C.I. = 0.97–0.99; p<0.001), indicating an inverse relationship between the amount of precipitation and the occurrence of WNV cases per county. The IRR represents the change in the risk of WNV infection for every one centimeter increase in total seasonal precipitation. As such, a one cm increase in total precipitation corresponds to a 2% decrease in the risk of WNV infection. Temperature was also associated with WNV incidence (IRR = 2.0; 95% C.I. = 1.81–2.21, p<0.001), with each one degree increase in temperature corresponding to a 100% increase in the risk of WNV infection. Finally, total hydrogeographic area was also associated with WNV incidence (IRR = 0.98; 95% C.I. = 0.97–0.99, p = 0.002). Here, each one square kilometer increase in hydrogeographic area corresponded to a 2% decrease in WNV risk.

**Table 1:** Unadjusted incidence rate ratios (IRR) for the crude bivariate associations between human WNV and the landscape variables.

Landscape factors	IRR	95% C.I.	p-value
Model 1			
Precipitation^1^ (cm)	0.98	0.97–0.99	<0.001
Model 2			
Temperature^2^ (°C)	2.0	1.81–2.21	<0.001
Model 3			
Hydrogeographic area (sq. km)	0.98	0.97–0.99	0.002

These associations are derived from Poisson models, with county and year as the panel levels. Model 1 is comprised of precipitation as the single independent variable, Model 2 is comprised of temperature as the single independent variable, and Model 3 is comprised of hydrogeographic area as the single independent variable.

1 Precipitation is the total monthly precipitation accumulation in centimeters from May through October.

2 Temperature is the monthly-weighted seasonal cumulative temperature in degrees Celsius.

When these relationships were considered together in the adjusted Poisson model, the associations remained between WNV and hydrogeographic area and between WNV and temperature, but not between WNV and precipitation (Table 2). For every one degree increase in temperature, the risk of WNV infection was more than doubled (IRR = 2.06; 95% C.I. 1.84–2.31, p<0.001), while for every one square kilometer increase in hydrogeographic area the risk of WNV infection decreased by 1% (IRR = 0.99; 95% C.I. 0.98–0.997, p = 0.04). Precipitation was no longer significantly associated with WNV infection after adjusting for hydrogeography and temperature. The Moran's index was used to identify global spatial clustering of the residuals from the full Poisson model. A separate index was calculated for each year under study. The indices varied only modestly between −0.018 and −0.02 from 2000 to 2010 and were highly non-significant (all p-values>0.95). As such, there did not appear to be any substantive spatial clustering over time.

**Table 2:** Adjusted incidence rate ratios (IRR) for the independent associations between human WNV and the landscape variables.

Landscape factors	IRR	95%. C.I.	p-value
Precipitation^1^ (cm)	1.005	0.99–1.01	0.16
Temperature^2^ (°C)	2.06	1.84–2.31	<0.001
Hydrogeographic area (sq. km)	0.99	0.98–0.997	0.04

The full model is derived from multiple Poisson regression, with county and year as the panel levels. The associations between WNV occurrence and the independent variables are adjusted for the other variables in the model.

1 Precipitation is the total monthly precipitation accumulation in centimeters from May through October.

2 Temperature is the monthly-weighted seasonal cumulative temperature in degrees Celsius.

Table 3 shows the results of the instrumental variable analysis used to identify a potential bird-mediated association between WNV infection in humans and water in the landscape. The number of dead infected birds was positively associated, as a mediator of hydrogeographic features and precipitation, with WNV human cases (β = 0.15; 95% C.I. 0.02–0.28, p = 0.02). For every seven infected birds identified, one human WNV case could be expected.

**Table 3:** Instrumental variable analysis showing the association between WNV human cases and WNV bird deaths with precipitation and hydrogeographic area as the instruments are presented below.

Mediator	Regression coefficient	95%. C.I.	p-value
Birds (# of dead birds positive for WNV)	0.15	0.02–0.28	0.02

This model is derived from instrumental variable regression for panel data, with county and year as the panel levels.

**Figure 1:**
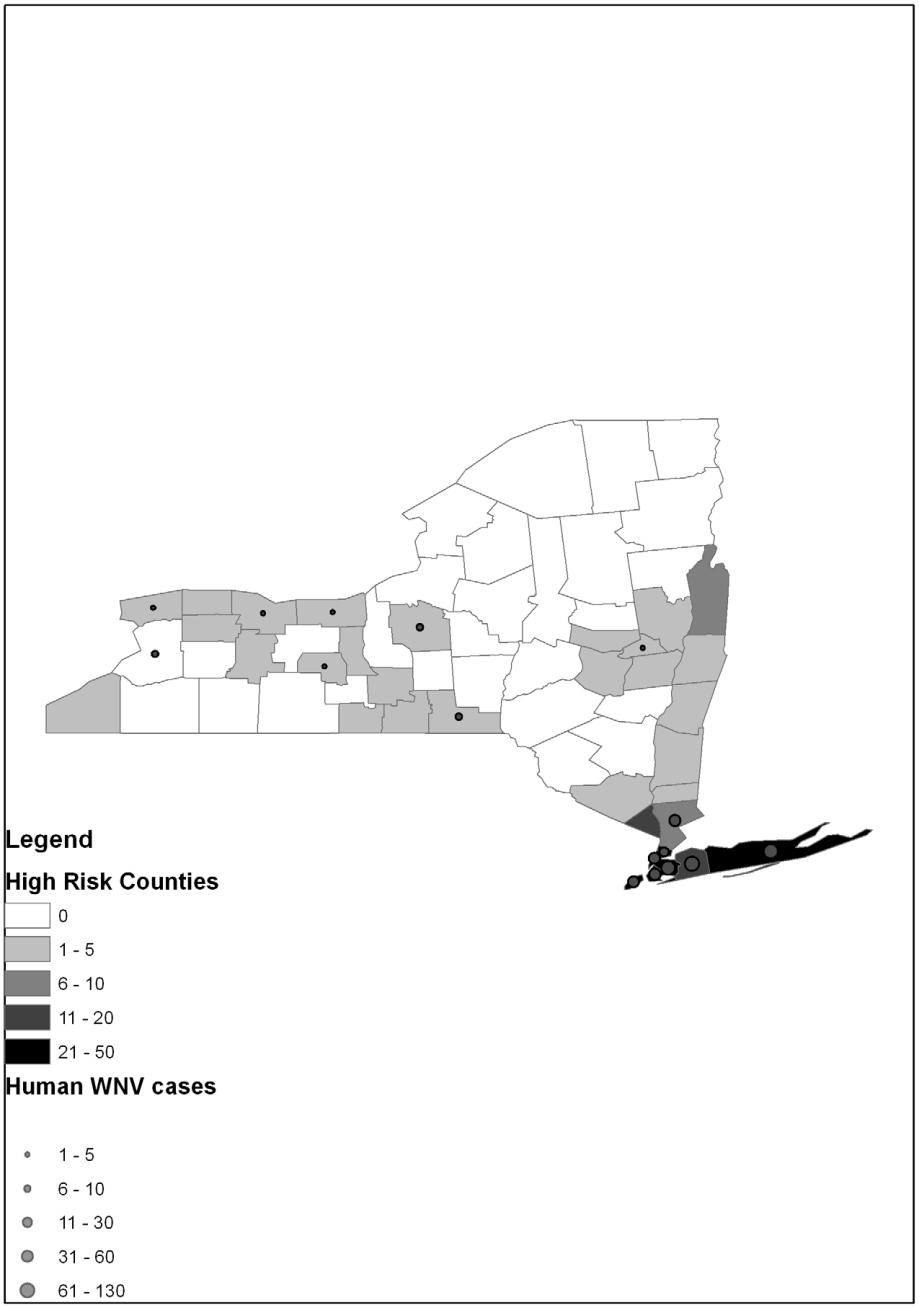
Map of the cumulative West Nile virus cases versus that which is predicted by climate and hydrogeography from the years 2000 to 2010. The number of human WNV cases as graduated symbols are superimposed over the monochrome choropleth of cases predicted by temperature, precipitation and hydrogeographic area.

Figure 1 shows graduated symbols, representing the occurrence of human cases of WNV, superimposed over a choropleth map of WNV cases predicted by precipitation, hydrogeography, and temperature. This map shows the clustering of observed human WNV cases along the Atlantic Ocean/Long Island Sound and Lake Ontario Tributaries watersheds in the southeastern and western coastal areas, respectively. Moreover, the predicted WNV cases match the observed counts in geographic distribution, with the preponderance of observed and predicted cases identified in these same watershed areas. However, the observed and predicted cases do deviated somewhat among the northern counties of the Hudson River Valley. Nevertheless, these counties did experience a high number of WNV among birds (data not shown in map).

## Discussion

This report found strong independent associations between WNV cases and both climatic and hydrogeographic factors across 11 years of observance in New York State. Specifically, increasing precipitation was significantly associated with decreasing WNV cases, but not after considering the amount of surface water in the landscape and temperature. Conversely, increasing hydrogeographic area was significantly associated with decreasing WNV cases independently of both precipitation and temperature. Temperature was consistently positively associated with WNV cases having adjusted for both precipitation and hydrogeography. Moreover, the association between water in the environment and human WNV infection may be mediated by birds during the same years. It should be stressed that these findings cannot be interpreted as causal, but they do suggest the possibility for an important relationship between hydrogeography and climate in the complex epidemiology of WNV infection.

The inverse relationship identified in this report between precipitation and WNV in humans has been documented before. Outbreaks are being increasingly identified in concert with dry seasons of low precipitation [9]–[10], [22]. Hot temperatures have also been identified previously with increased WNV incidence [8]. The current findings agree with previous results, as this report also found that increasing precipitation was associated with decreased WNV occurrence. However, this current report adds further nuance to this relationship by showing that the amount of precipitation accumulating transiently may be less important than the amount of surface water present. The importance of water is clear from the perspective of mosquito ecology. *Culex* mosquitoes transmit WNV in North America. *Culex pipiens* is the most important WNV vector in the northeastern United States in general, and in New York in particular. This mosquito, as with all mosquito vectors, requires water to complete its life cycle, and it prefers standing water containing organic material. The pregnant female ovipositions on the water's surface, and the larvae feed and develop under the water's surface. Subsequently they pupate and eventually emerge as adults [23]. While water is critical for the mosquito ecology, so too is it critical for *C. pipiens*' preferred hosts: passerine birds. These birds also require abundant water in the landscape for their survival. Because the passerine birds are the preferred hosts of the mosquito vectors in the northeast, the vectors do not typically pursue human hosts when these birds are present in adequate numbers [24]. Moreover, those periods where human infection does occur appear to coincide with periods of drought or diminished precipitation for specific geographic areas [9], [10]. It has been suggested that periods characterized by lower than normal precipitation may lead to increased human infection because passerine species are forced to find more reliable sources of water beyond their typical local geography [11]. Moreover, it has been shown that geographic areas demonstrating higher densities of dead crows correspond to areas of higher incidence of human infection [25], and that birds may facilitate the effects of climate on human infection [17]. In this report, while both precipitation and hydrogeographic area were individually associated with human WNV cases, precipitation was no longer associated when adjusted for hydrogeographic area. Moreover, the hydrogeography of the landscape was consistently associated with human WNV cases. If hydrogeography were to play a role in such an ecology, those landscapes with greater hydrogeographic area may be able to sustain local bird populations even during periods of low precipitation, which would subsequently provide *C. pipiens* with an adequate natural host population. On the other hand, those landscapes with less hydrogeographic area may be more sensitive to local precipitation. As such, periods of low precipitation may correspond to reduced (i.e. dispersed) bird populations, because there is less water in the landscape when there is less precipitation, and thus the need for *C. pipiens* to seek alternative hosts. This report cannot, however, support or refute this specific ecology as an important etiologic pathway in the landscape epidemiology of WNV. Nevertheless, this report does agree with the two studies that have shown hydrogeographic features to be important in determining *C. pipiens* abundance in the landscape and its capacity for West Nile transmission [13], [14]. The potential mediation of the association between human WNV infection and water in the environment by WNV-infected birds was assessed in this report. A significant association was identified between human WNV cases and infected birds treating precipitation and hydrogeographic area as instruments. While the association does identify a signal worth exploring with more localized field surveys of bird populations proximal to human cases, a moderated interpretation of the association is required for two important reasons. First, WNV-infected birds were obtained by passive surveillance across all counties. Only those birds that died, and only those birds that died in locations permitting their identification, could be included in ongoing bird surveillance. As such, there would have been many bird infections, likely the majority, that were not captured. Second, the number of infected birds are aggregated within counties. As such, the specific microgeographic features that may be relevant to bird infections, and which would also be relevant to their mediating potential between human WNV infections and water in the landscape, cannot be delineated within county and are instead dilute across these larger regions of space.

This study has some important limitations. The outcome and explanatory variables are all aggregated at the county level. As such, the specificity of the described associations is diminished and the resolution of the ecologic and epidemiologic picture is necessarily coarse. Undoubtedly, more localized, microgeographic phenomena with respect to water in the landscape will influence the transmission of human WNV, as well as modify the influence of birds as described above. Since this report did not address such localized features, the results are only suggestive, not conclusive. Furthermore, any direct causal claims would require a controlled experimental design involving randomized selection of mosquito and bird species georeferenced precisely with the hydrogeography of the landscape, and in close observation with the intra- and interseasonal variation in temperature and precipitation. Moreover, human cases of infection would also need to be georeferenceed in the same way such that the microgeographic covariation between these variables and human WNV infections may be better qualified to capture the true influence of these factors on human infection. Finally, the results described in this report are limited to the northeastern United States. It has been suggested that the virus, the relevant *Culex* and birds species, and the physical landscape, may converge toward very different disease ecologies in the western US [12]. As such, similar findings, though possible, should not necessarily be expected west of the Mississippi River.

In conclusion, this study identified a unique association between WNV occurrence and the hydrogeographic area of the landscape that is independent of precipitation and temperature. This is the first time this association has been documented as a potential component of the landscape epidemiology of WNV in the northeastern United States. This epidemiologic feature may result from aspects of bird ecology that affect mosquito host-seeking behavior, however, the data in the current study are too limited to posit a bird mediator in the causal pathway. In addition, given the nature of the county aggregated data, this investigator is wary of drawing any causal conclusions with respect to water in the landscape. Instead, this study serves as a surveillance instrument that has been used to identify a potentially important signal with respect to the hydrogeography and landscape epidemiology of WNV transmission. This report does not purport to define etiologic pathways in this transmission, but rather suggests follow-up surveys of mosquito and bird populations, and human WNV cases, in areas both proximal and distal to large bodies of water in the landscape.
